# A Comparison of Gene Expression Profiles between Glucocorticoid Responder and Non-Responder Bovine Trabecular Meshwork Cells Using RNA Sequencing

**DOI:** 10.1371/journal.pone.0169671

**Published:** 2017-01-09

**Authors:** Jaclyn Y. Bermudez, Hannah C. Webber, Bartley Brown, Terry A. Braun, Abbot F. Clark, Weiming Mao

**Affiliations:** 1 North Texas Eye Research Institute, University of North Texas Health Science Center, 3500 Camp Bowie Blvd. Fort Worth, TX, United States of America; 2 Stephen A. Wynn Institute for Vision Research, University of Iowa, Iowa City, IA, United States of America; 3 Department of Ophthalmology and Visual Sciences, Carver College of Medicine, University of Iowa, Iowa City, IA, United States of America; 4 Department of Biomedical Engineering, College of Engineering, University of Iowa, Iowa City, IA, United States of America; Universitat Regensburg, GERMANY

## Abstract

The most common ocular side effect of glucocorticoid (GC) therapy is GC-induced ocular hypertension (OHT) and GC-induced glaucoma (GIG). GC-induced OHT occurs in about 40% of the general population, while the other 60% are resistant. This study aims to determine the genes and pathways involved in differential GC responsiveness in the trabecular meshwork (TM). Using paired bovine eyes, one eye was perfusion-cultured with 100nM dexamethasone (DEX), while the fellow eye was used to establish a bovine TM (BTM) cell strain. Based on maximum IOP change in the perfused eye, the BTM cell strain was identified as a DEX-responder or non-responder strain. Three responder and three non-responder BTM cell strains were cultured, treated with 0.1% ethanol or 100nM DEX for 7 days. RNA and proteins were extracted for RNA sequencing (RNAseq), qPCR, and Western immunoblotting (WB), respectively. Data were analyzed using the human and bovine genome databases as well as Tophat2 software. Genes were grouped and compared using Student’s t-test. We found that DEX induced fibronectin expression in responder BTM cells but not in non-responder cells using WB. RNAseq showed between 93 and 606 differentially expressed genes in different expression groups between responder and non-responder BTM cells. The data generated by RNAseq were validated using qPCR. Pathway analyses showed 35 pathways associated with differentially expressed genes. These genes and pathways may play important roles in GC-induced OHT and will help us to better understand differential ocular responsiveness to GCs.

## Introduction

Glucocorticoids (GCs) are anti-inflammatory agents used to treat ocular diseases such as uveitis and macular edema. However, prolonged ocular application of GCs may lead to GC-induced ocular hypertension (OHT) and GC-induced glaucoma (GIG), a severe side effect that can lead to permanent visual loss. GC-OHT can also occur with other non-ocular routes of administration such as systemic application of GCs and endogenous elevation of cortisol that can lead to Cushing’s syndrome/disease, although the incidence of GC-induced OHT is lower than with topical GC application [1]. GIG is a secondary glaucoma, which is clinically and pathologically similar to primary open angle glaucoma (POAG) [2,3]. Prolonged ocular administration of GCs results in OHT in approximately 40% of the general human population [4–7]. The subjects who develop GC-induced OHT are considered GC responders, while those who do not develop OHT are considered non-responders. However, studies showed that over 90% of the POAG patients are GC responders, which is significantly higher than non-POAG individuals [7]. GC responders are at greater risk for developing POAG [7–9]. These studies further suggest the correlation between POAG and GIG.

One of the major risk factors associated with both GIG and POAG is elevated intraocular pressure (IOP). IOP elevation results from increased aqueous humor (AH) outflow resistance caused by damage to the trabecular meshwork (TM), a multilayered tissue that accounts for the majority of the AH drainage. GCs affect the TM by increasing its stiffness, causing cytoskeletal rearrangement, inducing excessive extracellular matrix deposition, and altering cell adhesion [3,10,11]. These alterations may contribute to IOP elevation and glaucoma pathogenesis.

Since GIG pathogenesis shares similar pathology to POAG, GIG has often been used as a tool to understand the molecular mechanisms of POAG. GC-induced OHT has been reported in several animal models including murine, rat, feline, leporine, ovine, bovine eyes [12–22]. A similar 40% responder rate was also seen in nonhuman primate eyes [15]. Overby and Zode each showed that C57BL/6J mice develop OHT after treatment with systemic or topical dexamethasone (DEX), respectively [23,24]. Rice and colleagues reported that only some mice on the mixed C57BL/6J-Tyr(c-Brd) x 129S5/SvEvBrd (B6.129) background developed elevated IOP, suggesting there may be mouse strain differences in GC responsiveness [19]. However, the GC responder rate in some models is different from that in human. For example, some studies showed that 100% of the cows and sheep that received topical prednisolone developed OHT [13,16].

In addition to in vivo animal models, ex vivo models are also useful tools for studying GIG. In contrast to the high cost, time, and limited availability of animals (especially primates and livestock), ex vivo models are relatively affordable and readily available. Perfusion cultured human eyes have long been used in GIG research [25–28]. The responder rate of perfusion cultured non-glaucomatous human eyes is very close to the observations in human subjects [25]. However, human donor eyes are prioritized for corneal transplantation, and the eyes available for research often have other ocular diseases or insufficient corneal endothelia. Due to these concerns, we developed a bovine anterior segment perfusion culture model for studying GIG [29]. Using this model, we found that bovine eyes have a similar responder rate to that of the general human population and human anterior segment perfusion cultures, showing that the bovine ex vivo GIG model is a suitable replacement/alternative to the human ex vivo model.

Although both in vivo and ex vivo GIG models enable researchers to monitor IOP changes, the yield of RNA or protein from TM tissues, especially small lab animals, is often insufficient for gene array or proteomic studies. In addition, the TM pigment content interferes with RNA purification, cDNA synthesis, and protein estimation [30]. Due to these reasons, cultured TM cells (in vitro models) are frequently used in screening/discovery studies. The major disadvantage of using TM cells is that the IOP and GC responsiveness of the eye from which the TM cells are isolated is usually unknown. Without this information, it is difficult to verify whether a TM cell strain is a responder or non-responder. The lack of GC responsiveness information may explain the inconsistency between several microarray studies [31–36].

In this study, we combined our bovine ex vivo and in vitro models to determine the genes that are differentially expressed in bovine TM (BTM) cells. Our study is unique because: 1) we used TM cells from eyes with known GC responsiveness and 2) we used RNA sequencing (RNAseq) to compare gene expression between GC responders and non-responders.

## Methods

### Bovine Anterior Segment Perfusion Culture

Paired bovine eyes were obtained from a local abattoir and transported to the laboratory on ice within six hours from time of sacrifice. One eye from each pair was subjected to ex vivo perfusion organ culture, while the fellow eye was used to establish the BTM cell strain. The perfusion culture procedure was previously described [29]. Briefly, the extraocular tissue was removed from the eyes, and the eyes were sterilized with Betadine (Purdue Products, Stamford, CT) for 2 minutes, followed by two rinses with PBS. The eyes were scored and dissected with scissors along the equator, and the posterior segment was discarded. We carefully removed the vitreous, uveal tract, and lens without disturbing the TM. The remaining anterior segment was then mounted on a custom made Plexiglass dish. A size-matched Plexiglass O-ring was then used to clamp the anterior segment against the Plexiglass dish at the equator with four plastic screws. This mounting created a water-tight artificial anterior chamber. Each Plexiglass dish had two embedded cannulas: one for medium infusion and the other for IOP measurement via a pressure transducer (ADInstruments, Colorado Springs, CO). DMEM-high glucose medium (Thermo Scientific, Waltham, MA) containing 1% glutamine, 1% penicillin + streptomycin, and 1% amphotericin B (Sigma-Aldrich, St. Louis, MO) was infused at a constant infusion rate of 5μL/min using a syringe pump (PHD2000; Harvard Apparatus, Holliston, MA). Pressure transducers were connected to a data acquisition system (PowerLab; ADInstruments) consisting of a signal amplifier, a bridge amplifier, and a computer with the LabChart software (ADInstruments). Stable baseline IOPs were established within the first 24 hours of bovine anterior segment perfusion. Eyes were then treated with 100 nM DEX initially dissolved in ethanol (EtOH) (Sigma-Aldrich). IOPs were recorded every minute. For data analysis, baseline IOP was defined as the average of IOP measured 12 hours prior to treatment. After treatment started, IOP was averaged every 24 hours. ΔIOP was defined as averaged IOP minus baseline IOP. Maximum ΔIOP (mΔIOP) was the highest IOP post-treatment and was used to determine DEX-responsiveness. Eyes with an mΔIOP equal or greater than 2.82 mmHg were considered responders and those below 2.82 mmHg were considered non-responders as previously described [29].

### Establishment of BTM Strains & Assessment of DEX Responsiveness

The contralateral eye of paired bovine eyes was used for BTM cell isolation and cell culture. The TM tissue was dissected under a dissection microscope, and placed into the well of a 24-well culture plate filled with DMEM-high glucose medium (Thermo Scientific) containing 1% glutamine, 1% penicillin + streptomycin, and 10% fetal bovine serum (FBS) (AtlasBiologicals, Fort Collins, CO). BTM cells migrated from the TM tissue onto the culture plate surface within a few days. BTM cells were passaged 1:2 to 1:3 from a 24-well plate to 12-well plate, 6-well plate, and T-25 flask. Confluent BTM cells were then treated with either 0.1% EtOH as a vehicle control or 100nM DEX for 7 days. To verify the identity of these TM cells, the formation of cross-linked actin networks (CLANs) as well as the expression of TM cell markers, including collagen IV, laminin, and α-smooth muscle actin were verified in all the BTM cells strains using immunofluorescent staining [37–39]. Conditioned medium was collected and used for Western immunoblotting (WB). After electrophoresis using 4–15% SDS-PAGE and transfer, the blots were blocked with 5% dry milk and incubated with the rabbit anti-fibronectin antibody (Millipore, Billerica, MA). The blots were washed and incubated with a secondary goat anti-rabbit antibody conjugated with HRP (Cell Signaling Technology, Danvers, MA). SuperSignal West Femto Maximum Sensitivity Substrate (Thermo Scientific) was used for signal detection and images were taken using the FluroChem^TM^ 8900 imager (Cell Biosciences, Santa Clara, CA). Some SDS-PAGE gels were stained with Coomassie blue (GelCodeBlue, Thermo Scientific) to ensure equal protein loading.

### RNA Extraction

Confluent BTM cells cultured in 6 well plates from established strains were treated with EtOH or DEX as previously described in DMEM+0.5% FBS for 7 days, and medium was changed every other day. Total RNA was extracted using the RNA purification kit (RNeasy Mini Kit, Qiagen) with DNase I treatment for 15 minutes. RNA was quantified using the NanoDrop 2000 (Thermo Scientific) and RNA integrity (RIN) was measured using the Agilent Bioanalyzer (Agilent Technologies). Bovine TM cell strains with an RIN >9.5 and a concentration of 100ng/μl or higher were used for RNA sequencing.

### Expression Profiling by RNASeq

Transcript profiling using the RNASeq was performed at the University of Iowa, Iowa Institute of Human Genetics, Genomics Division (Iowa City, IA). Briefly, 300 ng total RNA was sheared using the Covaris E220 (Covaris, Inc., Woburn, MA), converted to cDNA and ligated to sequencing adaptors containing indexes using the Illumina TruSeq stranded mRNA sample preparation kit (Cat. #RS-122-2101) following the manufacturer’s recommended protocol (Illumina, Inc., San Diego, CA). The resulting libraries were normalized by index, pooled, and sequenced using Illumina v3 (2 x 100 bp paired-end) sequencing chemistry run on an Illumina HiSeq 2000 (Illumina, Inc.). The data have been submitted to http://www.ncbi.nlm.nih.gov/sra (accession number PRJNA315985).

### Mapping and Expression Quantification

The UMD3.1 genome assembly of bovine was used for mapping and annotation. We used Tophat2 [40] to perform mapping, Cuffquant for quantitation, and Cuffnorm and Cuffdiff for normalization and differential expression analysis. The 10^th^ percentile of the level of expression was added to the fragments per kilobase of exon per million fragments mapped (FPKM) values reported by Cuffdiff to regularize the expression values. This diminishes artifacts of large or small fold change values as a result of a measured value for expression being close to zero.

To determine if the genes were both statistically and biologically significant, we performed a two-step analysis:

1. Selected the genes with the FDR-adjusted p-value (i.e. the q-value) < 0.05.
2. For the genes that already showed statistical significance (p<0.05), we further set a cutoff line of 20% change, i.e. only genes with ≥20% increase or decrease in expression were selected.

For DEG group #3, we performed a 3^rd^ analysis. We compared the increase or decrease between responders and non-responders. Only genes showing ≥20% difference in increase or decrease between responder vs. non-responders groups are listed. We included an example in S1 Appendix to clarify our analyses.

### Real Time qPCR

The same RNA samples used for RNAseq were used for reserve transcription. cDNA was synthesized using the iScript cDNA synthesis kit (Bio-Rad). In each qPCR test tube, 20 μl reaction mix was prepared using SSoAdvanced SYBR Green Supermix (Bio-Rad). qPCR was performed in the CFX96 thermocycler (Bio-Rad). The thermoprofile was 40 cycles of 95°C for 10 seconds and 60°C for 30 seconds, followed by a dissociation curve check. The PCR primers are listed in Table 1. The ΔΔCt method was used to calculate gene expression changes, and *actin* was used as an internal control.

**Table 1:** Primers for q-PCR.

Gene	Forward Primer	Reverse Primer
*DKK1*	Forward: ccttggatgggtactccaga	Reverse: gcacagtctgatgagcgaag
*HMGA2*	Forward: caagagtccctccaaagcag	Reverse: ttgtggccatttcctaggtc
*MT2A*	Forward: aaaggggcttcggacaagt	Reverse: ctatttacaccggggagcag
*C1QTNF7*	Forward: gatggtagagacggcaggaa	Reverse: caggaggccctacttctcct
*CDH6*	Forward: tgaggctggatacagtgcag	Reverse: ccaacccaaaagagaagcaa
*SPARCL1*	Forward: ccaatcagatgctgttttgga	Reverse: ctcggctaccgtgttcaagt
*ALOX12*	Forward: cattggacgtgttccagaga	Reverse: ggtaacccttccttccaggt
*CYYR1*	Forward: ttgctcagtgtggcaaagac	Reverse: gggtggtgccagaaagaata
*RMRP*	Forward: tgctgaaggcctgtttccta	Reverse: cagggtaggatcgcttcttg
*CCL5*	Forward: cgctttggagttgagctagg	Reverse: agagcgagaagcaaagttgg
*IFI6*	Forward: actcgttggcctcctcact	Reverse: agaaaggccccgatcttg
*IFI27*	Forward: gaatcactgcctcctccttg	Reverse: cccaccaagagtttggatga
*S100A12*	Forward: gctgaagcagctgatcacaa	Reverse: tctttatcggcatccaggtc
*SLC2A5*	Forward: agtctcctggcaaacgaaga	Reverse: aagaagggcaggaagaggag
*PTX3*	Forward: catatgccagttgggaaggt	Reverse: gccttctccagtctcccttt
*AANAT*	Forward: cgagaggccttcatctctgt	Reverse: aagtctttcctcgtcccaca
*CRABP1*	Forward: cacgaccgagatcaacttca	Reverse: cccctccagaagagtttgtg
*PTHLH*	Forward: aataagtccccagagcgaga	Reverse: gctccattgctgaactagcc
*Actin*	Forward: ctcttccagccttccttcct	Reverse: gggcagtgatctctttctgc

### Pathway and Protein-protein Interaction Analysis

Pathways associated with genes identified by RNAseq were analyzed using the WEB-based GEne SeT AnaLysis Tool kit (WebGestalt) (http://bioinfo.vanderbilt.edu/webgestalt/) [41,42]. We identified the genes whose expression was at least 20% different between the GC responder and non-responder BTM cell strains. User data and parameters: User data: textAreaUpload.txt, Organism: homosapiens, Id Type: gene_symbol, Ref Set: entrezgene, Significance Level: Top10, Statistics Test: Hypergeometric, MTC: BH, Minimum: 2.

## Results

### Establishment of BTM Responder and Non-Responder Cell Strains

**Fig 1:**
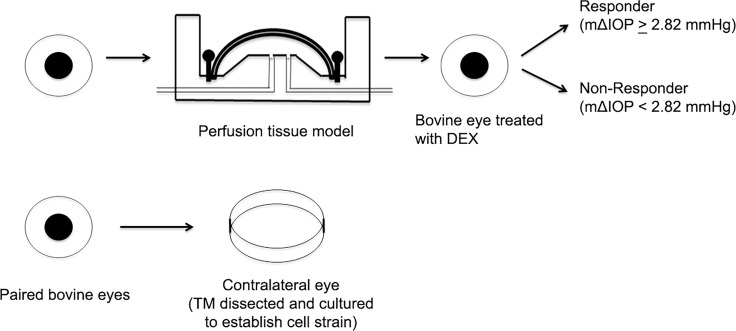
Establishment of responder and non-responder BTM cell strains. One of the paired bovine eyes was used for perfusion culture, treated with DEX, and IOP was monitored. The fellow eye was directly used to establish a cultured BTM cell strain without prior perfusion culture. Based on the DEX-induced IOP changes in the perfusion cultured eyes (mΔIOP ≥2.82mmHg or <2.82mmHg), the BTM cell strains established from the fellow eyes were defined as responder cell strains or non-responder cell strains, respectively.

Three GC responder and non-responder BTM cell strains were established using the approach described in “Methods.” Only confluent BTM cell cultures were used to mimic in vivo conditions [37,43]. Fig 1 demonstrates how BTM cell strains were categorized. One bovine eye was used for perfusion culture and DEX treatment, while the fellow eye was used for BTM cell establishment. According to the IOP response to DEX in the perfused eye, the BTM cell strain established from the fellow eye was defined as a responder or non-responder strain using our established criteria [29]. In the present study, we perfused 26 pairs of bovine eyes, and found that 7 pairs were responders with a responder rate of 36.8% which is very close to our published study [29]. DEX-induced OHT usually developed with 3–5 days. The mΔIOP of our responder cell strains selected for RNAseq ranged from 3 to 5.46 mmHg, while the mΔIOP of non-responder cell strains were no more than 1.53 mmHg (Table 2).

**Table 2:** The IOP change in the fellow eye of DEX responder (R) and non-responder (N) TM cell strains.

Cell Strain	mΔIOP mmHg
BTM 56 Responder	3.19
BTM 61 Responder	4.43
BTM 64 Responder	5.46
BTM 73 Non-Responder	0.74
BTM 80 Non-Responder	0.50
BTM 81 Non-Responder	1.53

**Fig 2:**
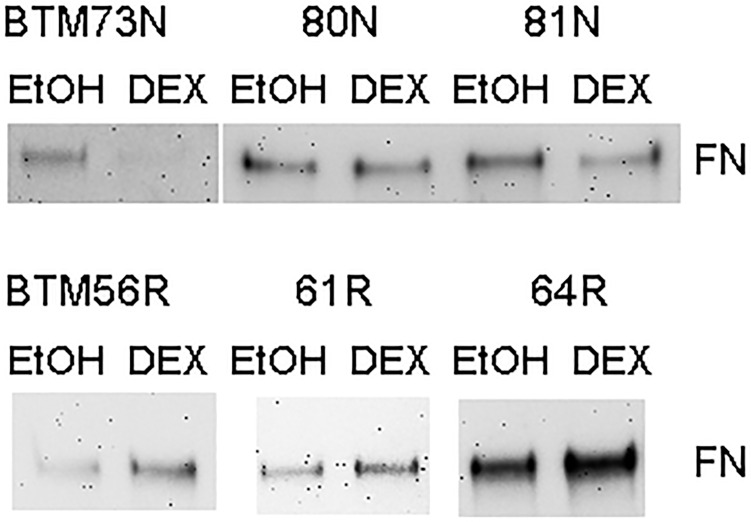
Differential induction of Fibronectin (FN) by DEX in responder and non-responder BTM cells. Confluent BTM cells were treated with 0.1% EtOH or 100nM DEX for 7 days. Conditioned medium was collected for WB. R: responder BTM cells. N: non-responder BTM cells.

We then treated the responder and non-responder BTM cells with 0.1% EtOH or 100nM DEX for 7 days and collected conditioned medium for WB. We found that all 3 responder cell strains showed an induction of fibronectin, a GC-inducible protein, while the 3 non-responder cell strains did not (Fig 2 and S1 Fig).

### RNAseq Showed Differential DEX-Induced Gene Expression between BTM Responder and Non-Responder Cell Strains

**Fig 3:**
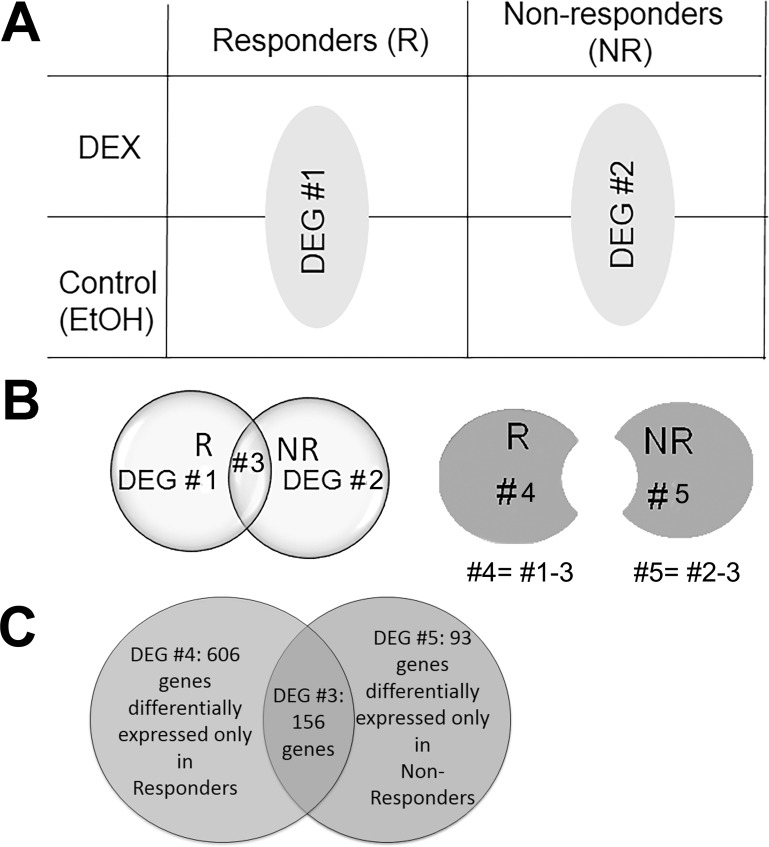
Diagram of differential expression groupings (DEG). The four groups of raw data (responders and non-responder BTM cells treated with DEX or EtOH) were grouped into 5 DEGs. A) The initial grouping of raw data. DEG #1: DEX vs. EtOH in responders; DEG #2: DEX vs. EtOH in non-responders. B) Further grouping of DEGs 3–5. DEG #3: overlap between DEG groups #1 and 2; DEG #4 = 1–3; DEG #5 = 2–3. C) The number of genes in DEGs#3, 4, and 5.

The 6 BTM cell strains (3 responder and 3 non-responder) were treated with either EtOH or DEX for 7 days. RNA was extracted, analyzed for quality and quantity, and used for RNAseq library preparation. After expression quantitation, differential gene expression analysis was carried out using the differential expression groupings (DEG) strategy (Fig 3). Expression values are reported in fragments per kilobase of transcript per million fragments mapped (FPKM), as described by Trapnell et al [40].

The definition of individual DEG groups:

DEG #1: Differentially expressed genes between DEX vs. EtOH treated responder cells.
DEG #2: Differentially expressed genes between DEX vs. EtOH treated non-responder cells.
DEG #3: The overlap between DEG groups #1 and #2 (S1 Table)
DEG #4: DEG #1 (responder changes) minus DEG #3 (S2 Table).
DEG #5: DEG #2 (non-responder changes) minus DEG #3 (S3 Table).

**Fig 4:**
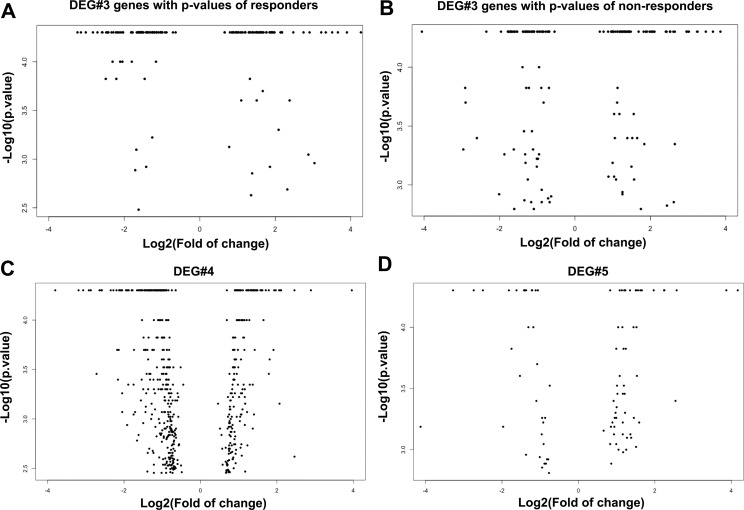
Volcano plot of DEGs 3–5. The fold of change (log_2_) and p value (-log_10_) of the genes in DEGs 3, 4, and 5 are shown in volcano plots. Since the genes in DEG#3 have two p values, one from responders and the other from non-responders (S3 Table), they are shown in two plots, (A) and (B), respectively. (C) DEG#4; (D) DEG#5.

RNAseq showed genes were differentially expressed in the presence of DEX between responder and non-responder groups: 156 genes in DEG #3, 606 genes in DEG #4, and 93 genes in DEG #4 (all with p values <0.05, n = 3). Interestingly, all the genes in DEG#3 showing up or down-regulation in the responder group had the same trend of regulation (up or down-regulation) in the non-responder group. The fold change and p value of the genes in DEGs 3–5 are shown in Fig 4.

### qPCR Validation

**Fig 5:**
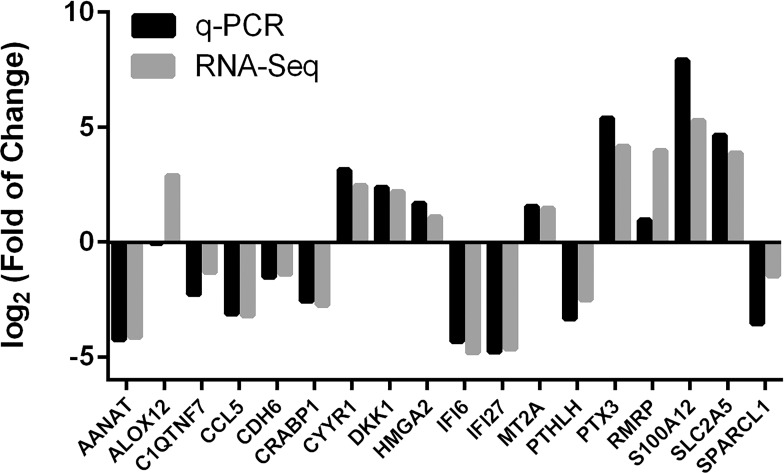
Validation of RNA sequencing findings using qPCR. The same RNA used for RNAseq was used for qPCR. The ΔΔCt method was used for calculation of gene expression changes and actin was used as an internal control. Data analysis/grouping was performed in a similar way as shown in Fig 3. Three of the most up-regulated and down-regulated genes of DEG groups 3, 4, and 5 were studied and compared to RNAseq results. Values of Log_2_(Fold of change): >0: up-regulation; = 0 non change; <0 down-regulation. n = 3.

We used qPCR to validate the expression of 3 of the most up-regulated and 3 of the most down-regulated genes in DEGs 3, 4 and 5 (total 18 genes, Table 3) identified by RNAseq. cDNA was prepared using the same RNA that was used for RNAseq. Since *GAPDH* showed significant changes to DEX treatment in the responder group (S2 Table), *actin* was used as an internal control. Our qPCR data closely matched RNAseq data (Fig 5) except for *ALOX12*, confirming the reliability of RNAseq technique.

**Table 3:** Three of the most up-regulated and down-regulated genes in DEGs 3–5.

Gene	DEG	Up or Down Regulated	Fold of change
*DKK1*	3	Up	4.55
*HMGA2*	3	Up	2.12
*MT2A*	3	Up	2.75
*C1QTNF7*	3	Down	0.40
*CDH6*	3	Down	0.38
*SPARCL1*	3	Down	0.36
*ALOX12*	4	Up	7.42
*CYYR1*	4	Up	5.52
*RMRP*	4	Up	15.57
*CCL5*	4	Down	0.11
*IFI6*	4	Down	0.04
*IFI27*	4	Down	0.04
*S100A12*	5	Up	39.16
*SLC2A5*	5	Up	14.62
*PTX3*	5	Up	17.98
*AANAT*	5	Down	0.06
*CRABP1*	5	Down	0.15
*PTHLH*	5	Down	0.18

### Pathway Analysis

**Fig 6:**
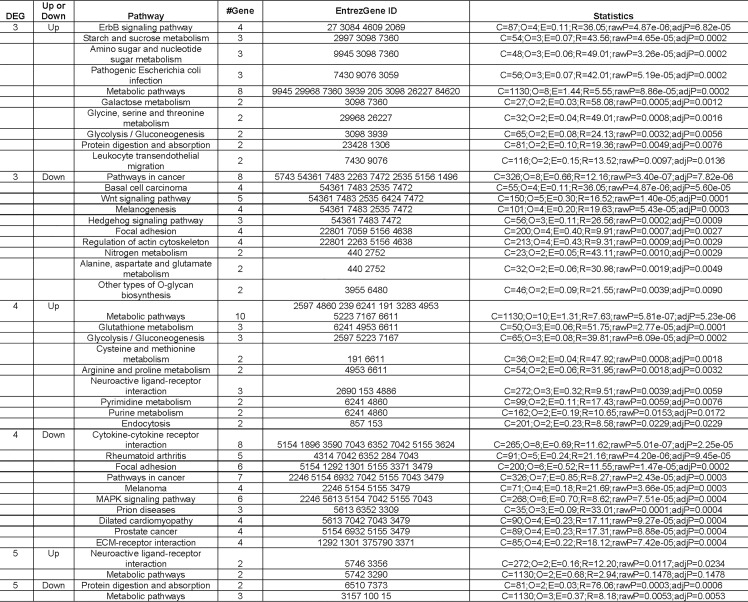
Pathways associated with DEGs 3, 4, and 5. C: the number of reference genes in the category; O: the number of genes in the gene set and also in the category; E: the expected number in the category; R: ratio of enrichment; rawP: p value from hypergeometric test; adjP: p value adjusted by the multiple test adjustment. Please be aware that “Up or Down” only refers to whether the genes associated with listed pathways were up or down-regulated. It does not necessarily mean the pathway was activated or inhibited.

We used the WebGestalt tool to determine the biological pathways associated with DEGs 3, 4 and 5 (Fig 6). Our analysis identified 35 pathways which may play important roles in differential GC-responsiveness.

## Discussion

We used the highly sensitive RNAseq technique to compare DEX-responder and non-responder bovine TM cells. Different pools of genes were cross-compared, and a number of genes were found to be differentially expressed between responder and non-responder BTM cells. Pathway analyses showed that 35 pathways were closely associated with DEX responsiveness. Our results showed that GC-responder and non-responder TM cells react differently to GCs.

Several studies explored DEX-induced gene expression changes in the TM using microarray techniques [31–36]. Although many genes were found to be differentially expressed upon DEX treatment, there was little consistency among those reports (Summarized in Table 4), which is very likely due to the use of TM cells of unknown GC-responsiveness. In contrast, our study is the first that has compared gene expression between TM cells isolated from eyes with known IOP and GC responsiveness.

**Table 4:** Summary of differential microarray gene expression studies in TM cells/tissues.

Sample Type	Microarray Chip Type	Reported Genes Upregulated	Reference
Human TM cells	Micromax, Perkin-Elmer	myocilin (*MYOC*), decorin, insulin-like growth factor binding protein 2, ferritin L chain, and fibulin-1C	[32]
Human TM cells and Optic nerve head astrocyte cells	U95Av2 GeneChips, Affymetrix	TIGR/MYOC, a serine protease inhibitor (alpha1-antichymotrypsin), a neuroprotective factor (pigment epithelium-derived factor), an antiangiogenesis factor (cornea-derived transcript 6), and a prostaglandin synthase (prostaglandin D(2) synthase)	[31]
Human TM cell line	MicroMax Human cDNA System I, Perkin-Elmer	GAS1, CDH4, MT1L, CST3, ATF4, ASNS/TS11, CHOP, HSPA5	[33]
Human TM cells	U133A Gene Chip, Affymetrix	SLP1, SAA2, ANGPTL7, MYOC, SAA1, SERPINA3, ZBTB16	[30]
Human TM cells	Coated human cDNA microarrays (UltraGAPS; Stanford FunctionalGenomics Facility)	MYOC, MT2A, GAS1, MT1G, CSNK1G2,MT1F, SF1, MT1L, IRF7, AGXT, DNA2L, and MED6	[29]
Human TM Cells	Human Whole Genome Oligo, Agilent	RGC32, OCA2, ANGPTL7, MYOC, FKBP5, SAA1 and ZBTB16	[34]
Bovine TM Tissues	GeneChip Bovine Genome Array; Affymetrix	KCNMB1,ITGA8, DES,PLN, ACTA2, RBM24, PTPRR, COL24A1, CNN1, AGT, SMTN, RASL12, TGFB1I1, CD55, CKB, MRVI1, PCP4L1, HSPB8, TAGLN	[53]

Our data revealed differentially expressed genes that are involved in cell-adhesion, metabolism, extracellular matrix, and inflammatory response. Among these genes, we are particularly interested in *Dickkopf 1* (*DKK1*) and *K-Cadherin* (*CDH6*). *DKK1* is an inhibitor of the Wnt signaling pathway. We have previously reported that the Wnt pathway plays a role in regulating IOP in perfusion cultured human eyes and the mouse eye [43,44]. We also found that inhibition of the Wnt signaling pathway by *DKK1* increased IOP [43]. One potential mechanism for this increase is through the stiffening of the trabecular meshwork [45,46]. Also, inhibition of the canonical Wnt signaling may promote ECM deposition [47]. In human osteoblasts, Ohnaka and colleagues found that *DKK1* is up-regulated by GCs [48,49]. *DKK1* is also increased in the extracellular matrix of DEX treated TM cells [45]. Therefore, *DKK1* and the associated Wnt signaling, may play important roles in ocular GC responsiveness. *SFRP1* is another Wnt pathway inhibitor, and we found that it is elevated in the glaucoma trabecular meshwork (GTM) and is able to induce OHT in mouse as well as human eyes [44].

In contrast to *DKK1*, the potential role of *CDH6* in glaucoma pathogeneses is currently unclear. However, our preliminary studies suggest that CDH molecules may be involved in IOP regulation. *CDH6* is expressed in the human trabecular meshwork (HTM), and our unpublished data show that *CDH6* is able to inhibit *SFRP1*-induced OHT in mouse eyes. We believe that *CDH6* and other cadherin molecules maintain TM homeostasis, and the disruption of these molecules contributes to OHT. Our hypothesis is supported by our findings that *CDH6* was down-regulated by DEX treatment in both responder and non-responder TM cells, but the expression of this gene was more suppressed in responders (DEG 3).

Besides Wnt and cell adhesion pathways, several well characterized pathways were also identified in this study. It is not surprising to find that the metabolic pathways of hexoses, polysaccharides and amino acids were among those pathways since they are known to be regulated by GCs. We also found cytokine and ECM related pathways are differentially regulated by GCs between responder and non-responders. Cytokines, especially Interleukin-6 (*IL-6*), have been extensively studied. *IL-6* is induced by mechanical stress-induced *TGFβ1* expression [50,51]. Liton and colleagues showed that *IL-6* lowers outflow resistance in perfusion cultured porcine anterior segments, suggesting this cytokine may play a role in maintaining normal IOP, although Birke and colleagues found no hypotensive effects using a similar model [50,52]. We found that a number of inflammatory response-related molecules such as *IL11/27RA*, *CXCL3* and *CCL2/5*, *as well as IF16*, *IFI27* were down-regulated in the responder group (DEG 4, S2 Table). In contrast, pro-inflammatory proteins *IL-6* and *S100A12*, were up-regulated by more than 1.5 or 39 fold, respectively, in the non-responder group (DEG 5, S3 Table). The difference in ILs and other inflammation associated molecules may contribute to the difference in IOP between the two groups.

Another group of important molecules revealed by this study were molecules involved in ECM turnover. Numerous studies have shown that there is excessive ECM deposition in GIG and POAG TM cells and tissues. We found that both responder and non-responder groups had a down-regulation of *MMP12*, but this down-regulation in the responder group was much more than that in the non-responder group. In fact, *MMP12* is the most down-regulated gene in DEG 3 (S1 Table). In addition to *MMP12*, up-regulation of *TIMP1* and down-regulation of *MMP3* were observed in response to DEX treatment in the responder group (S2 Table), but not in the non-responder group. MMPs and their inhibitors TIMPs work together to maintain TM ECM and IOP [53–56]. The dysregulation of *MMP3*, *MMP12* and *TIMP1* in the responder TM may provide further clues in explaining differential IOP responses.

In addition to the pathways and genes that have known implications in the TM and POAG, the expression of many genes are altered by GCs in other tissues, but have not been reported in the POAG TM. For example, *HMGA2*, a member of the high mobility group AT-hook protein family that participates in DNA-protein interaction, plays a key role in chromatin architecture and gene regulation [57]. Overexpression of *HMGA2* is associated with many types of tumors [57]. In POAG, loss of TM cells and fibrotic changes are observed, and TM cell senescence [58] may exacerbate these changes. The DEX induced up-regulation of *HMGA2* may be a compensatory mechanism to antagonize TM cell senescence. Metallothionein 2A (*MT2A*), a metal binding enzyme, plays a role in anti-oxidant, anti-apoptosis, detoxification and anti-inflammation [59]. It is also a GR-inducible gene in hepatic cells [60]. We found that *MT2A* is one of the most up-regulated genes in DEG#3 (Table 3), suggesting *MT2A* may be one of the factors that mediates GC responsiveness. Secreted protein acidic and rich in cysteine like protein 1 (SPARCL1) is a matrix protein whose level is associated with tumor metastasis and prognosis [61]. Naschberger et al. showed that SPARCL1 contributes to tumor endothelial cell quiescence [61]. In POAG TM, Rhee and colleagues found that SPARC plays a role in IOP regulation and TM pathology [62]. Although we found a DEX-induced down-regulation of SPARCL1 in responders, it may indicate a remodeling of the TM. Alternatively, the BTM may rely on different proteins compared to the HTM. Arachidonate 12-Lipoxygenase (ALOX12) metabolizes arachidonic acid as well as other lipids, and generates reactive oxygen species (ROS) [63]. Although the TM has a powerful system to handle oxidative stress throughout its lifespan [64], DEX-induced ALOX12 expression may increase the ROS burden of TM cells and therefore accelerate TM damage. The RNA component of mitochondrial RNA processing endoribonuclease (RMRP) gene is an untranslated gene. The transcript of the RMRP gene is a component of an RNA-dependent RNA polymerase complex consisting of RMRP RNA and TERT [65]. This complex is important for miRNA processing as well as cell and mitochondrial functions [65–67]. A DEX-induced increase in RMRP will likely to affect TM mitochondrial functions and inhibit a large number of genes [67]. The solute carrier family 2 member 5 (*SLC2A5*) gene encodes the fructose transporter GLUT5, and the aralkylamine N-acetyltransferase (AANAT) gene encodes an enzyme that plays a role in melatonin synthesis. Increased GLUT5 is found in tumor cells as a feature of their metabolism [68], while changes in AANAT is associated with depression [69]. Until now, most of the research of GIG are at cellular and molecular biology levels. The changes in SLC2A5 and AANAT suggest that the difference between GC responders and non-responders in their biochemistry is worthy of further investigation. Pentraxin 3 (*PTX3*) is a member of the pentraxin protein family. *PTX3* is an inflammatory marker that has been found in immune cells and vascular cells [70]. However, many studies showed this protein may have a protective role on inflammation in the cardiovascular system [71,72]. In a clinical study, Lerzo and colleagues showed that DEX prophylaxis elevates *PTX3* levels in pediatric patients receiving open heart surgeries, suggesting DEX induced *PTX3* may contribute to decreased inflammation as well as improvement in prognosis [73]. Our data showed that *PTX3* is one of the most upregulated genes in DEG5 (Table 3), indicating *PTX3* may also protect the TM and prevent OHT in the non-responder eyes. The parathyroid hormone-like hormone (*PTHLH*) gene is a member of the parathyroid hormone family. PTHLH regulates endochondral bone development and epithelial mesenchymal interaction. Flöttmann et al. reported that duplication of *PTHLH* causes osteochondroplasia [74]. Since Borras and colleagues suggest that the pathological changes in the POAG TM resembles calcification [75], a down-regulation of *PTHLH* in the non-responder group may help to explain why their TM is less effected by DEX. The cellular retinoic acid binding protein 1 (CRABP) is involved in retinoic acid signaling. The role of retinoic acid in fibrosis is controversial [76]. Our findings that DEX selectively decreased CRABP1 in the non-responder group may suggest a profibrotic role of CRABP1 and retinoic acid in the TM. Further investigation of these genes previously not reported as being DEX responsive in the TM warrants further investigation.

In another study using the in vivo bovine model, Danias and colleagues collected BTM tissues from cattle treated with or without topical prednisolone, and used bovine microarray to compare gene expression [77]. However, in that study, the authors reported a 100% responder rate and genes identified showed little overlap with our current data. We believe that the difference in cattle strains and GCs contribute to the discrepancy between the two studies. Also, the bovine genome continues to be updated. The genes detectable in microarrays vary based on how frequently the manufacturer updates their arrays. In contrast, RNAseq allows us to observe and map transcripts to the most up-to-date bovine assembly. Another advantage of RNAseq is its much wider dynamic range that enables the detection of transcripts expressed at very high and very low levels. In principle, RNAseq can detect the vast majority of RNA transcripts, including noncoding RNA.

In addition to the discovery of GIG related genes and pathways, our study provided useful technical information. For qPCR and microarray studies, a number of housekeeping genes are used as internal controls. However, the choice of housekeeping genes under various situations is often overlooked. We found that the *GAPDH* gene showed a 1.68 fold increase in response to DEX in the responder group but not in the non-responder group (S2 and S3 Tables). Since the RNAseq data are normalized using the FPKM method (see Methods) that does not reply on a single gene but the overall readings, we believe that our observation of differential expression of *GAPDH* is reliable. Therefore, we used *actin* as the internal control for our qPCR study instead of *GAPDH* since the expression of *actin* was not affected by DEX in either the responder or the non-responder groups (S2 Appendix). This selection may also be suitable for GC-related studied in HTM cells/tissues.

Although this study provided useful observations with respect to gene differential expression in GC responders and non-responders, there are still some unsolved questions. We were unable to detect significant DEX-induced *myocilin* (*MYOC*) expression, while we previously reported DEX-induced MYOC expression in conditioned medium from perfusion cultured bovine eyes [29]. Our unpublished data also showed that DEX rarely induces *MYOC* at protein or mRNA levels in BTM cell cultures, suggesting the expression *MYOC* is affected by adjacent tissues/environments in the bovine eye. Similarly, no DEX-induced *MYOC* expression in BTM cell cultures was reported in two other studies using BTM cells [43,78]. We previously showed that the induction of *MYOC* by DEX in HTM cells is a secondary, indirect response, requiring the DEX-induced synthesis of another protein [79]. We hypothesize that BTM cells may have a different *MYOC* induction pathway than HTM cells. Also, we were unable to compare the ratio of glucocorticoid receptor α (*GRα*) to *GRβ* due to the unavailability of bovine *GRβ* sequence. *GRα* is the functional GR receptor while the alternatively spliced *GRβ* acts as a dominant negative form isoform. The ratio of *GRα* to *GRβ* is a mechanism that may contribute to ocular GC-responsiveness [10,80]. Nevertheless, our RNAseq data suggest a potential alternative splicing site at the 3’ end of *GRα* which may help us to identify *GRβ* (data not shown). Although we did not observe DEX-induced *MYOC* expression, we found CLAN formation in confluent cell cultures, which is a characteristic of TM cells. We also observed DEX-induced expression of caveolin 1 (*CAV1*), a GC- inducible gene. Aga and colleagues found that knockdown of *CAV1* increases outflow facility in perfusion cultured human eyes [81]. In our study we found that *CAV1* was up-regulated in DEG 4 (responder only genes), and this upregulation may contribute to decreased outflow facility and OHT.

In contrast to *MYOC*, we observed a clear difference in DEX-induced fibronectin expression using WB. Fibronectin is an important component of the ECM. In addition to mechanical support, it functions as a reservoir for many growth factors. For example, TGFβ2, a POAG-associated factor, binds to the latent TGF-β binding protein (LTBP). This protein complex anchors to fibronectin and other ECM molecules [82]. During tissue mechanical deformation or damage, there is a release of the active form of TGFβ2 which activates the TGFβ pathway [83]. Accumulation of excessive fibronectin is believed to increase outflow resistance in the TM and elevates IOP. Many studies showed that glaucoma-associated factors, including GCs, are able to induce fibronectin [10,38]. We found that the induction of fibronectin correlated well with BTM responsiveness and IOP elevation, and our findings suggest that fibronectin induction may be one of the contributing factors to GC-induced OHT. Interestingly, transcription factor binding analysis (http://www.sabiosciences.com/chipqpcrsearch.php?species_id=0&factor=GR-beta&gene=fn1&nfactor=n&ninfo=n&ngene=n&B2=Search) shows that there are no GC response elements (GRE; GR binding sites) in the fibronectin gene, like the *MYOC* gene [79]. It is very likely that GC-induced fibronectin is a secondary response. Besides, there is a discrepancy between mRNA and protein levels of fibronectin. In contrast to DEX-induced fibronectin in conditioned medium collected from the responder group (Fig 2), the mRNA expression ratios of fibronectin did not show significant difference in either responders or non-responders (S2 Appendix). Since RNA and conditioned medium were collected simultaneously from the same cell cultures, we believe that this difference is likely due to post-translational processing. There are several possibilities: 1) microRNA (miRNA). DEX may induce miRNAs that affect fibronectin translation in non-responders since miRNA may repress translation without degrading mRNA; 2) protein cross-linking. It is well recognized that increased ECM protein cross-linking may slow down protein turnover. In this study, we found lysyl oxidase-like 2 (LOXL2), an enzyme that cross-links ECM proteins, is more elevated in the responder group (S1 Table); 3) protein degradation. As discussed previously, we found that MMP12 which degrades fibronectin, is significantly decreased in responders (S1 Table). Also, MMP3 is selectively decreased while TIMP1 is selectively increased in responders, but not in non-responders upon DEX treatment (S2 Table).

In conclusion, we developed an approach to establish bovine TM cell cultures with known GC responsiveness. Combined with the powerful RNAseq technique, we discovered a number of genes and pathways that may mediate differential GC responsiveness in the eye. Further studies are needed to determine the exact function of each gene/pathway in GC-induced OHT and GIG.

## Supporting Information

S1 Appendix Determination of DEG#3. (DOCX)

S2 Appendix The expression levels of fibronectin and actin. (XLSX)

S1 Fig Coomassie blue staining of conditioned medium. Equal amount of conditioned medium was separated on 4–15% SDS-PAGE gradient gel as described in Fig 2. The gels were stained with Coomassie blue to show total proteins.
(DOCX)

S1 Table DEG#3. (XLSX)

S2 Table DEG#4. (XLSX)

S3 Table DEG#5. (XLSX)
