# Supplementary material for: A Comparison of Gene Expression Profiles between Glucocorticoid Responder and Non-Responder Bovine Trabecular Meshwork Cells Using RNA Sequencing
Source: PLoS One. 2017 Jan 9;12(1):e0169671. doi: 10.1371/journal.pone.0169671 (PMC5222504; doi:10.1371/journal.pone.0169671)
Supplement: S1 Appendix — (DOCX) [file pone.0169671.s001.docx]

**Determination of DEG3**

DEG1 is the genes showing significant changes in the responder group (Dex treated vs. EtOH treated samples); DEG2 is the genes showing significant changes in the non-responder group (Dex treated vs. EtOH treated samples).

For example:

If DEG1 has 5 genes: A (2 fold increase), B (4 fold increase), C (2 fold decrease), D (4 fold decrease), G (0.5 fold decrease)

And DEG2 has 5 genes: A (0.5 fold increase), E (1.5 fold increase), C (0.5 fold decrease), D (4 fold decrease), F (1 fold decrease)

Then our results would yield the following DEGs:

DEG3: A, C

DEG4: B, G

DEG5: E, F

Gene D is considered insignificant because it showed the same amount of down-regulation in responders and non-responders. Therefore, gene D is not included in DEGs 3, 4, or 5.
