# Supplementary material for: A Comparison of Gene Expression Profiles between Glucocorticoid Responder and Non-Responder Bovine Trabecular Meshwork Cells Using RNA Sequencing
Source: PLoS One. 2017 Jan 9;12(1):e0169671. doi: 10.1371/journal.pone.0169671 (PMC5222504; doi:10.1371/journal.pone.0169671)
Supplement: S1 Fig — Equal amount of conditioned medium was separated on 4–15% SDS-PAGE gradient gel as described in Fig 2. The gels were stained with Coomassie blue to show total proteins. (DOCX) [file pone.0169671.s003.docx]

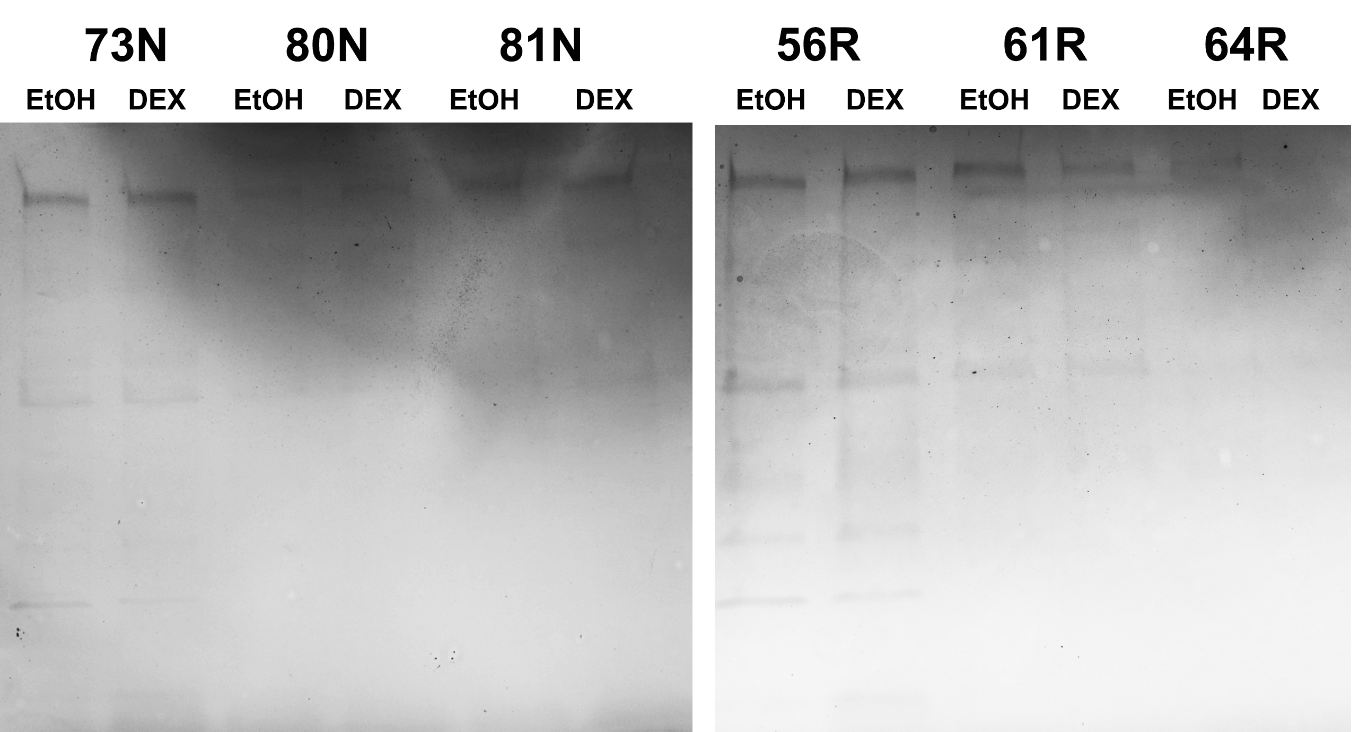


Supplemental Figure 1. Coomassie blue staining of conditioned medium

Equal amount of conditioned medium was separated on 4-15% SDS-PAGE gradient gel as described in Figure 2. The gels were stained with Coomassie blue to show total proteins.
